# Editorial: The biological pump: a hunt for microbial key players involved in ocean carbon and nutrient fluxes

**DOI:** 10.3389/fmicb.2026.1818353

**Published:** 2026-03-30

**Authors:** Taichi Yokokawa, Daniele De Corte, Abhishek Srivastava

**Affiliations:** 1 Institute for Extra-cutting-edge Science and Technology Avant-garde Research (X-star), Japan Agency for Marine-Earth Science and Technology (JAMSTEC), Yokosuka, Japan; 2 Tohoku University & JAMSTEC Advanced Institute for Marine Ecosystem Change (WPI-AIMEC), Yokohama, Japan; 3 National Oceanography Centre, Southampton, United Kingdom; 4 The Department of Functional and Evolutionary Ecology, University of Vienna, Vienna, Austria

**Keywords:** carbon transformations, microbial loop, organic matter (DOC), oxygen-deficient zones (ODZs), particulate organic carbon (POC), the biological carbon pump

The Earth's oceans cover ~71% of the planet's surface and account for approximately 97% of the world's free water (Durack, 2015). The ocean supports a wide range of life forms whose growth and sustenance are regulated by nutrient availability (Fernández-González et al., 2022). This Research Topic comprises 10 articles that advance our understanding of the functioning of the complex oceanic biological carbon pump (BCP), addressing the relationship between living microbes and their surrounding environment. These microbes transform organic matter and recycle nutrients across diverse oceanic realms.

The BCP is often viewed as a direct pathway for exporting carbon from the euphotic zone to the deep ocean; however, it consists of a series of complex processes that are shaped by microbial activity (Siegel et al., 2023). One of the key factors regulating carbon transfer into the dark ocean is the production of organic carbon through photosynthesis (Ducklow et al., 2001). Current models that link cellular carbon content to chlorophyll a are widely used to estimate phytoplankton biomass and, consequently, the available carbon content. Gui and Sun highlighted the usefulness of these models, their potential applications, and the challenges of their future development. Furthermore, the study by Alothman et al. revealed that environmental conditions also regulate the BCP. For example, in the warm, oligotrophic systems of the Red Sea, bacteria play a role in transforming organic matter and transferring carbon through the microbial loop to higher trophic levels. At later stages of organic matter degradation under anaerobic conditions, methanogenesis becomes a significant process, influencing whether carbon is buried in sediments or re-enters the ocean–atmosphere system as the greenhouse gas methane. The BCP currently stores thousands of petagrams of carbon in the ocean interior (Nowicki et al., 2022). Therefore, quantifying how much carbon is retained in deep waters and sediments and how much is recycled in the upper ocean, with additional loss of carbon as CO_2_ and CH_4_, is crucial to link the BCP to ocean biochemistry and improve climate change forecasts.

Four additional studies (by Liu and Huang, Padaki et al., Kim et al., and Ebihara et al.) published in this research topic highlighted how microbial processes influence the BCP at different ocean depths. These studies investigate carbon transformation in the surface layer, remineralization in the mesopelagic layer, and organic matter utilization in the bathypelagic layer. Ebihara et al. demonstrated the utility of extracellular polymeric substances in shaping the bacterial community in the mixed layers of the euphotic zone in the western North Pacific subtropical region. Padaki et al. focused on the surface ocean by characterizing diel patterns of volatile organic compound (VOC) production in a model diatom. They showed that VOC production is tightly linked to cellular metabolism and the cell cycle. Their results revealed that phytoplankton release of labile organic carbon is dynamically structured on a diel timescale, providing a mechanistic basis for how primary producers regulate carbon availability to heterotrophic microbes and potentially influence air–sea carbon exchange. Liu and Huang investigated temperature-dependent oxygen utilization rates in the mesopelagic waters in Southeast Asian seas, providing a quantitative assessment of oxygen utilization in relation to physical and biogeochemical controls. By integrating tracer-based water age estimates with Arrhenius-type reaction kinetics, their study demonstrated a robust positive relationship between temperature and oxygen consumption rates in the mesopelagic zone. This work refines our understanding of carbon turnover in the mesopelagic waters and highlights the sensitivity of mesopelagic respiration to ocean warming. Kim et al. expanded our understanding of microbial carbon cycling by culturing and sequencing the genome of a novel deep-sea *Burkholderiales* strain isolated from a depth of 4,000 m. This study emphasizes the importance of culture-based approaches in elucidating the functional capabilities of deep-sea microorganisms.

Three other studies (Huanca-Valenzuela et al., Zeng et al., and Engel et al.) addressed oxygen-deficient zones (ODZs) and the metabolic strategies of microbes associated with sinking particles as they transit through hypoxic and oxygenated waters. Huanca-Valenzuela et al. have shown the importance of sinking marine particles of various sizes that pass through zones with varying oxygen concentrations near the East Pacific Rise. Bacterial density was shown to increase on large sinking particles characterized by decreasing total organic carbon content with increasing depth. The authors concluded that both the ODZ and the plume region promote a chemosynthetic lifestyle. Zeng et al. emphasized the importance of studying hypoxic micro-niches where anaerobic processes may occur. In the low-oxygen intermediate waters of the South China Sea, they identified a high potential for denitrification, a process that is largely associated with anaerobic conditions. An elevated level of suspended particulate matter (SPM) and particulate organic carbon (POC) was positively related to increased denitrification potential, highlighting the role of terrestrial-derived SPM as a hotspot for coupling microscale carbon and nitrogen cycles. Engel et al. highlighted the role of sinking POC and microbe-mediated dissolved organic carbon diagenesis in deeper waters off the coast of Chile. Increased cell-specific extracellular enzyme activities, along with an elevated proportion of bacterial activity at depth, provide evidence of microbial adaptation to a low-substrate environment. An additional finding of this study, which was conducted at the Humboldt upwelling system site, was the enrichment of glycine (Gly) below the mesopelagic zone. The authors suggest that this enrichment may be linked to the distinct hydrography of the upwelling system, which contributes to the ventilation of the local oxygen minimum zone.

In summary, microbes transform and channel nutrients to sustain their growth while simultaneously modifying the local environments in which they live. These dynamic interactions between living cells, nutrients, and organic matter occur throughout all layers of the ocean, forming a fundamental component of the biological carbon pump. Furthermore, the studies included in this Research Topic also illustrate the close relationship between the BCP and other biogeochemical cycles, emphasizing their integral role in maintaining efficient ocean functioning.
